# Acquired Coagulation Factor XIII Deficiency With Spontaneous Splenic Rupture: A Case Report

**DOI:** 10.1002/ccr3.72030

**Published:** 2026-02-11

**Authors:** Jun Lu, Xijun Zhu

**Affiliations:** ^1^ Hematology Department Xuancheng People's Hospital Xuancheng Anhui P.R. China

**Keywords:** diagnosis, factor XIII deficiency, hemorrhage, prophylaxis

## Abstract

Coagulation factor XIII deficiency (FXIIID) is a rare hemorrhagic disease, mainly manifested as skin ecchymosis and hematoma. Because of its atypical clinical manifestations and normal results of routine coagulation test, platelet count and function, it has brought great challenges to the diagnosis. This case report introduces the diagnosis and treatment of an elderly male patient with FXIIID in detail, and aims to emphasize the necessity of detecting FXIII activity when spontaneous bleeding occurs in patients with normal routine coagulation function and platelet count. At the same time, it emphasizes the importance of etiology finding and individualized treatment and management for patients with FXIIID.

## Key Clinical Message

In FXIII‐deficiency, normal PT/APTT can mask life‐threatening bleeding; add FXIII assay in unexplained hematomas and pre‐operatively to avert catastrophic events like spontaneous splenic rupture.

## 1 Introduction

Coagulation factor XIII deficiency (FXIIID) is a rare bleeding disorder characterized by an impaired ability to stabilize fibrin clots. It leads to abnormal bleeding tendencies, including hematomas, as well as life‐threatening hemorrhages such as intracranial hemorrhage and umbilical stump bleeding [1, 2, 3]. This condition can manifest as either a hereditary deficiency, which follows an autosomal recessive inheritance pattern, or as an acquired deficiency resulting from specific underlying conditions, such as autoimmune disorders or liver disease [4, 5]. Although the exact prevalence of FXIIID is not well established, it is recognized as an infrequent disorder in the general population [6]. The estimated prevalence is approximately 1 in 2 million individuals, with many cases remaining underdiagnosed due to atypical presentations and the limitations of standard coagulation tests [2].

The clinical diagnosis of FXIIID faces two main challenges. First, normal results in standard coagulation tests (PT/APTT) often lead to missed diagnoses, necessitating specific FXIII testing for confirmation [7, 8]. Second, acquired cases are rare and must be differentiated from congenital forms. These cases require comprehensive genetic testing (F13A1/F13B negative), autoantibody screening, and evaluation of clinical characteristics for etiological classification. This classification directly influences treatment choices—simple replacement therapy versus combined immunosuppression—and impacts surgical risk stratification [9, 10, 11]. Therefore, including patients with unexplained bleeding in FXIII screening is crucial to avoid catastrophic outcomes.

In conclusion, this case report emphasizes the importance of recognizing FXIII deficiency as a potential cause of unexplained bleeding and highlights the critical need for tailored management strategies to address these specific issues. The complexities surrounding the diagnosis and treatment of FXIII deficiency serve as a reminder of the necessity for heightened clinical awareness and vigilance in managing patients with unexplained bleeding tendencies.

## 2 Case History/Examinations

The patient is a 71‐year‐old male, retired, who was admitted to the Hematology Department of the First Affiliated Hospital of the University of Science and Technology of China on September 17, 2024, with a right lower limb hematoma persisting for over 20 days and left upper limb skin bruising lasting 2 weeks. The patient has no obvious history of trauma.

Upon admission, a physical examination revealed a soft, purple hematoma approximately 3 × 4 cm in size on the lateral aspect of the right thigh. It was non‐tender. Additionally, several superficial ecchymoses were observed on the left upper extremity. No lymphadenopathy was detected in the superficial lymph nodes of the cervical, axillary, or inguinal regions, and the liver and spleen were not enlarged on palpation. Laboratory tests, including a complete blood count and a coagulation profile, showed no abnormalities, and renal and liver function tests were within normal limits. Further assessment of coagulation factors II, V, VII, VIII, IX, X, XI, and XII showed normal activity levels (Table 1). However, because of the patient's history of abnormal bleeding, additional coagulation factor tests including factors XIII and von Willebrand factor were conducted.

**TABLE 1:** Blood analysis.

Check item	Patient result	Reference ranges
PT	10.9 s	8–14 s
INR	0.93	
APTT	26.1 s	25–31.3 s
Fg	3.04 g/L	1.7–4 g/L
TT	18.2 s	14–21 s
D‐Dimer	0.4 μg/mL	0.01–0.55 μg/mL
FDP	2.5 μg/mL	0–5 μg/mL
AT‐III	85.4%	75%–125%
Platelet count (×10^9^/L)	311 × 10^9^/L	125–350 × 10^9^/L
Factor II activity	71.4%	65%–125%
Factor V activity	103.8%	65%–125%
Factor VII activity	111.6%	55%–170%
Factor X activity	83.9%	65%–125%
Factor VIII activity	84.6%	60%–150%
Factor IX activity	109.7%	60%–150%
Factor XI activity	100.0%	60%–150%
Factor XII activity	82.1%	50%–150%
vWF:Ag	136%	50%–160%
AFP/CEA/SCC/PSA	Negative	Negative
ANA	Negative	Negative
Positive screening test for FXIII deficiency	Fibrin clot dissolved within 26 min	Insolubilization

Abbreviations: AFP, alpha‐fetoprotein; ANA, antinuclear antibodies; APTT, activated partial thromboplastin time; CEA, carcinoembryonic antigen; FDP, fibrin degradation product; Fg, fibrinogen; PSA, prostate‐specific antigen; PT, prothrombin time; SCC, squamous cell carcinoma; TT, thrombin time.

## 3 Differential Diagnosis/Investigations/Treatment

We conducted a preliminary screening for coagulation factor XIII deficiency using the uric acid dissolution method, and the results showed complete dissolution of fibrin at 26 min, indicating a positive test for deficiency. The qualitative test result for coagulation factor XIII inhibitor was negative. Quantitative FXIII activity assays were unavailable at our institution during the study period, which represents a diagnostic limitation. Therefore, based on these tests, the patient was diagnosed with coagulation factor XIII deficiency. To classify the deficiency, whole‐exome sequencing was performed with complete coverage of F13A1 and F13B genes (no pathogenic variants detected). In addition, the peptide profiles of antinuclear antibodies (ANA), and extractable nuclear antigens (ENA) were negative, and tumor markers were normal. So far, we were unable to determine whether the patient had congenital or acquired FXIII deficiency.

Although we do not have clear evidence of immune factors, it is more likely to consider immune related acquired FXIIID in combination with the patient's age at onset. The patient received infusions of cryoprecipitate and fresh frozen plasma, and empirically attempted to take some immunosuppressive drug therapy consisting of prednisone, 20 mg orally, once daily, and cyclosporine capsules, 100 mg orally, twice daily. Following these interventions, the patient's bleeding symptoms improved, leading to the patient's discharge.

On October 26, 2024, the patient was readmitted to the Hematology Department of Xuancheng People's Hospital due to a large hematoma in the left hip. He received 20 U of cryoprecipitate and 400 mL of fresh frozen plasma, which led to the gradual resolution of the hematoma. On November 12, 2024, he visited the Hematology Department of Ruijin Hospital in Shanghai for a follow‐up consultation. Qualitative testing for coagulation factor XIII activity showed complete normalization within 1 h. Additionally, tests for protein C and protein S activities were within normal reference ranges, and platelet function tests revealed no abnormalities. Rheumatic and immune‐related tests indicated slightly elevated levels of anti‐phosphatidylserine IgM antibodies at 41.5 U/L, the clinical significance of which remains unclear (Table 2). We diagnosed the patient as possibly having acquired FXIIID, and the patient continued to receive oral cyclosporine and oral corticosteroid treatment.

**TABLE 2:** Blood analysis.

Check item	Patient result	Reference ranges
Protein C activity (%)	124%	70%–140%
Protein S activity (%)	96.7%	60%–130%
IgG	9.82 g/L	8.60–17.40 g/L
IgA	2.62 g/L	1.00–4.20 g/L
IgM	0.69 g/L	0.30–2.20 g/L
Anti‐ds‐DNA	Negative	Negative
CCP	< 0.5 RU/mL	0–25 RU/mL
ANCA‐PR3	0.18 RU/mL	< 20 RU/mL
ANCA‐MPO	0.70 RU/mL	< 20 RU/mL
Antiphosphatidylserine‐IgM	41.5 U/L	0–30 U/L

Abbreviations: A‐CCP, anti cyclic citrulline peptide antibody; ANCA‐PR3, antineutrophil cytoplasmic antibody target antigen PR3; ANCA‐MPO, antineutrophil cytoplasmic antibody target antigen MPO.

## 4 Outcome and Follow‐Up

**FIGURE 1:**
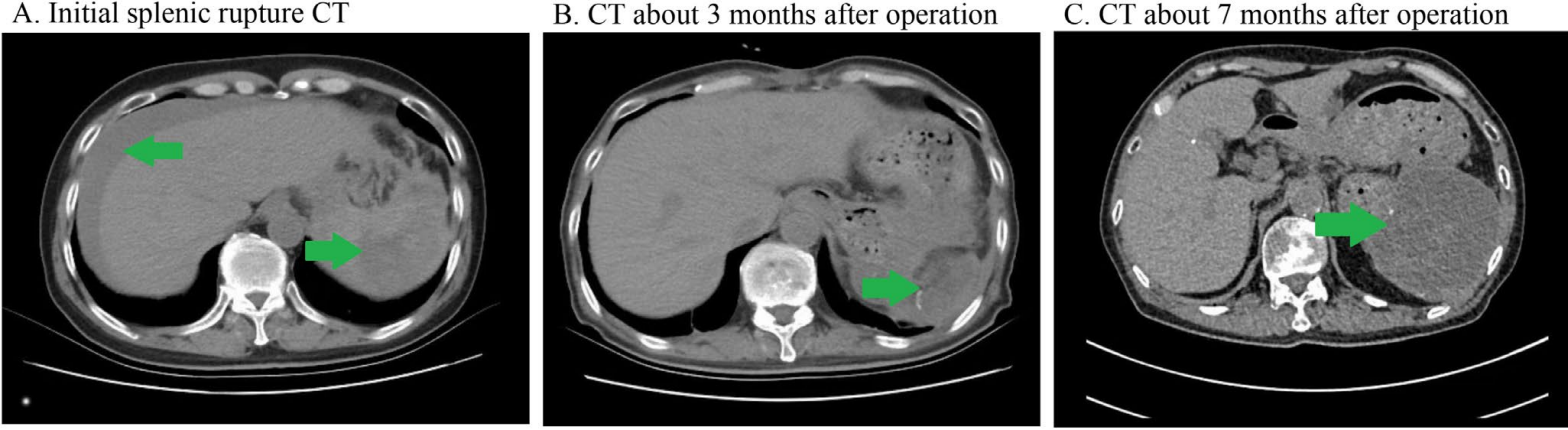
(A) The CT image of spontaneous splenic rupture. (B) The abdominal CT image at discharge after surgery. (C) Re‐examination of abdominal CT images after discharge for nearly 3 months.

On December 4, 2024, the patient developed sudden left abdominal pain with hemoglobin 71 g/L and hypotension (85/56 mmHg). Emergency CT confirmed spontaneous splenic rupture (Figure 1A). He received 20 units of cryoprecipitate preoperatively.

### 4.1 Surgery

Open splenectomy was performed due to hemodynamic instability and massive hemorrhage. Blood loss: > 5000 mL; autologous transfusion 4000 mL + RBC concentrate 1200 mL.

### 4.2 Intraoperative Coagulation Monitoring

Fibrinogen, bleeding condition, blood pressure, D‐dimer and thromboelastography (TEG) guided transfusion Postoperative transfusions: Daily FFP (400 mL) and cryoprecipitate (10 U) for 7 days, then weekly.

The patient experienced impaired wound healing with recurrent serosanguinous discharge from the surgical incision, characteristic of FXIIID‐related fibrin instability. He developed intra‐abdominal infections requiring broad‐spectrum antibiotics. Over 6 months, he received supportive therapy with albumin, FFP, and cryoprecipitate (Figure 1B). The incision healed slowly, and drains were removed by May 30, 2025.

Post‐discharge, he continued cyclosporine (100 mg BID) and prednisone (20 mg daily). Weekly cryoprecipitate prophylaxis (10 U) was maintained. PET‐CT showed no malignancy. Rituximab (100 mg/m^2^) was added weekly for 4 weeks starting June 16, 2025, to target autoimmune‐mediated FXIII consumption. Follow‐up FXIII activity assays remain pending due to regional unavailability. The patient continues to have intermittent skin hematomas and gradual left abdominal hematoma enlargement (Figure 1C), but no further life‐threatening bleeding.

## 5 Discussion

This case underscores the critical limitation of standard coagulation tests (PT/APTT) in detecting FXIIID [7, 8]. The diagnosis is established using a qualitative clot solubility assay; however, the unavailability of quantitative FXIII activity assays at our institution represents a significant diagnostic gap. Accurate activity measurement is essential for guiding prophylaxis dosing and monitoring treatment response, and its absence limits the generalizability of our management protocol.

The classification as acquired autoimmune FXIIID is supported by three key factors: (1) advanced age of onset (71 years), (2) negative whole‐exome sequencing of F13A1/F13B genes, and (3) presence of anti‐phosphatidylserine IgM antibodies. While the clinical significance of these antibodies remains unclear, their presence aligns with the autoimmune hypothesis [4, 8]. Acquired FXIIID is mediated by inhibitory autoantibodies that accelerate FXIII clearance, requiring immunosuppressive therapy rather than simple replacement [4, 9].

Congenital FXIIID presents in childhood with umbilical stump bleeding and has a clear genetic basis [2, 3]. In contrast, acquired forms manifest later in life and are associated with autoimmunity, malignancy, or liver disease [4, 5]. Our patient's age, negative genetic testing, and autoimmune marker positivity strongly favor an acquired etiology. The therapeutic implication is that without immunosuppression, factor replacement alone may be insufficient due to ongoing antibody‐mediated consumption. Due to the limitation of our test conditions, it is impossible to carry out the detection of factor XIII antibody and activity, which limits our accurate judgment of the curative effect of patients.

Perioperative management of FXIIID requires maintaining FXIII activity > 5%–10% to prevent bleeding [9, 12]. We used cryoprecipitate and FFP guided by TEG. The decision to perform open splenectomy was dictated by hemodynamic instability; laparoscopic surgery would have been preferable in a stable patient to reduce wound complications.

The impaired wound healing observed is mechanistically linked to FXIII's role in cross‐linking extracellular matrix proteins and promoting tissue repair [13, 14]. First, the core mechanism of the structural defect and premature dissolution of the fibrin clot is that the lack of FXIII leads to insufficient cross‐linking of fibrin, the clot presents a loose network structure, and the mechanical strength decreases by 60%–70% [15, 16]. Uncrosslinked fibrin is highly sensitive to plasmin, and α 2‐antiplasmin cannot be effectively anchored to fibrin, resulting in excessive activation of the fibrinolytic system [17]. Secend, FXIIIA not only crosslinks fibrin, but also catalyzes the covalent binding of a variety of matrix proteins. When FXIIIA is deficient, the extracellular matrix structure is fragile, unable to effectively support the migration of fibroblasts and endothelial cells, and the formation of granulation tissue is delayed [18]. Last, FXIIIA crosslinks matrix proteins under endothelial cells through transglutaminase activity and reduces vascular permeability. Surgical trauma leads to a physiological increase of local vascular permeability when FXIII is deficient, the repair of the endothelial barrier is blocked, and the protein rich liquid continues to leak into the incision space to form a vicious cycle of “seepage edema hypoxia,” which inhibits fibroblast function. This complication, while expected, highlights the need for intensive postoperative factor support.

Our immunosuppressive regimen (corticosteroids, cyclosporine, rituximab) was chosen to suppress B‐cell autoantibody production [9]. Rituximab was added after initial response was suboptimal. Serial FXIII activity and inhibitor titers (Bethesda assay) should be monitored to assess response, but were unavailable—an important limitation.

We advocated that although PT/APTT was normal, routine FXIII screening, quantitative activity assay and inhibitor testing should be carried out for all hematomas of unknown cause to make a definite diagnosis. Early immunosuppression and multidisciplinary preoperative planning for acquired cases were very important. Spontaneous splenic rupture is exceedingly rare in FXIIID, with only a few reported cases [9, 19], making this report clinically significant.

## 6 Conclusion

Acquired autoimmune FXIIID should be considered in elderly patients with unexplained bleeding and negative genetic testing. Quantitative FXIII activity measurement is essential but may be regionally unavailable, representing a diagnostic challenge. Multimodal therapy combining factor replacement and immunosuppression is required, though outcomes depend on antibody titer and treatment response. This case highlights the need for increased clinical awareness and improved diagnostic access.

## Author Contributions

**Jun Lu:** conceptualization, data curation, formal analysis, methodology, visualization, writing – original draft, writing – review and editing. **Xijun Zhu:** methodology, writing – review and editing.

## Funding

The authors have nothing to report.

## Ethics Statement

This research is observational in nature, thus does not necessitate ethical clearance.

## Consent

The patient and his children agreed to disclose their condition and signed a written informed consent.

## Conflicts of Interest

The authors declare no conflicts of interest.

## Data Availability

The data that support the findings of this study are available on request from the corresponding author. The data are not publicly available due to privacy or ethical restrictions.
